# Zbtb16 increases susceptibility of atrial fibrillation in type 2 diabetic mice via Txnip-Trx2 signaling

**DOI:** 10.1007/s00018-024-05125-2

**Published:** 2024-02-13

**Authors:** Zhi-Xing Wei, Xing-Xing Cai, Yu-Dong Fei, Qian Wang, Xiao-Liang Hu, Cheng Li, Jian-Wen Hou, Yu-Li Yang, Tai-Zhong Chen, Xiao-Lei Xu, Yue-Peng Wang, Yi-Gang Li

**Affiliations:** 1 Department of Cardiology, Xinhua Hospital Affiliated to Shanghai Jiao Tong University School of Medicine, 1665 Kongjiang Road, Shanghai, 200092 China; 2 Department of Cardiology, The Second Affiliated Hospital of Zhejiang University School of Medicine, Hangzhou, Zhejiang China; 3 Department of Biochemistry and Molecular Biology, Department of Cardiovascular Medicine, Mayo Clinic, Rochester, MN USA

**Keywords:** Zbtb16, Type 2 diabetes mellitus, Atrial fibrillation, Oxidative stress

## Abstract

Atrial fibrillation (AF) is the most prevalent sustained cardiac arrhythmia, and recent epidemiological studies suggested type 2 diabetes mellitus (T2DM) is an independent risk factor for the development of AF. Zinc finger and BTB (broad-complex, tram-track and bric-a-brac) domain containing 16 (Zbtb16) serve as transcriptional factors to regulate many biological processes. However, the potential effects of Zbtb16 in AF under T2DM condition remain unclear. Here, we reported that db/db mice displayed higher AF vulnerability and Zbtb16 was identified as the most significantly enriched gene by RNA sequencing (RNA-seq) analysis in atrium. In addition, thioredoxin interacting protein (Txnip) was distinguished as the key downstream gene of Zbtb16 by Cleavage Under Targets and Tagmentation (CUT&Tag) assay. Mechanistically, increased Txnip combined with thioredoxin 2 (Trx2) in mitochondrion induced excess reactive oxygen species (ROS) release, calcium/calmodulin-dependent protein kinase II (CaMKII) overactivation, and spontaneous Ca^2+^ waves (SCWs) occurrence, which could be inhibited through atrial-specific knockdown (KD) of Zbtb16 or Txnip by adeno-associated virus 9 (AAV9) or Mito-TEMPO treatment. High glucose (HG)-treated HL-1 cells were used to mimic the setting of diabetic in vitro. Zbtb16-Txnip-Trx2 signaling-induced excess ROS release and CaMKII activation were also verified in HL-1 cells under HG condition. Furthermore, atrial-specific Zbtb16 or Txnip-KD reduced incidence and duration of AF in db/db mice. Altogether, we demonstrated that interrupting Zbtb16-Txnip-Trx2 signaling in atrium could decrease AF susceptibility via reducing ROS release and CaMKII activation in the setting of T2DM.

**Graphical Abstract:**

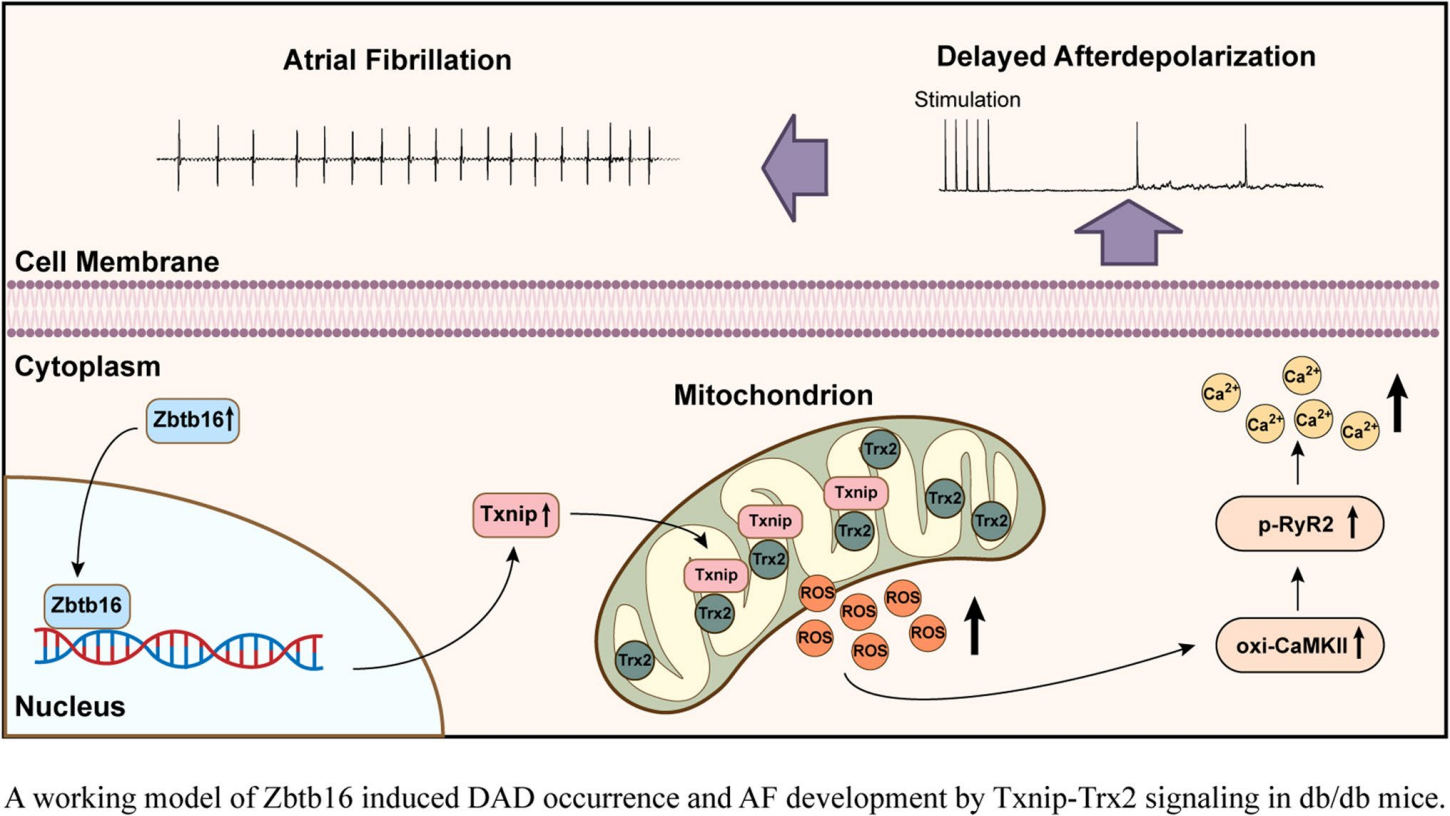

**Supplementary Information:**

The online version contains supplementary material available at 10.1007/s00018-024-05125-2.

## Introduction

Atrial fibrillation (AF) is the most prevalent sustained cardiac arrhythmia with typical clinical symptoms [1]. In recent studies, lifestyle-related conditions have been reported to contribute to the development of AF. Among them, patients with type 2 diabetes mellitus (T2DM), which accounts for more than 90% to 95% of the diabetes population, displayed at least twofold higher prevalence of AF [2–4]. However, the underlying mechanisms regarding the impact of T2DM on AF have not been fully elucidated.

RNA sequencing (RNA-seq) analysis in our study identified zinc finger and BTB (broad-complex, tram-track and bric-a-brac) domain containing 16 (Zbtb16) as the most significantly enriched gene in the atria of db/db mice compared to heterozygous littermates. Zbtb16 belongs to the Zbtb family and serves as an evolutionary conserved transcriptional factor [5]. Besides, Zbtb16 is known to be expressed in spermatogonial stem cells, hematopoietic stem cells, and neural progenitors and capable of regulating the development and effector function [6]. However, the potential effects of Zbtb16 in cardiovascular system are scarce now. Here, we combined RNA-seq with Cleavage Under Targets and Tagmentation (CUT&Tag) assay and identified thioredoxin interacting protein (Txnip) as the key downstream gene of Zbtb16. Txnip is considered as a critical regulator of cellular redox system since it can combine with the thioredoxin (Trx) which is a thiol-disulfide oxidoreductase and plays an important role in the reactive oxygen species (ROS) scavenging process. Once Txnip combines with Trx, the antioxidant function of Trx is inhibited [7]. In cardiomyocytes, there are two isoforms of Trx, including Trx1 and Trx2. Trx1 is mainly located in cytoplasm and Trx2 is only distributed in mitochondrion [8, 9]. In the present study, we verified Txnip was upregulated and mainly combined with Trx2 in mitochondria of db/db mouse atrial myocytes, which could lead to increased ROS release compared to db/ + littermates.

AF is closely associated with increased oxidative stress and ROS generation [10, 11]. Excess ROS release activates calcium/calmodulin-dependent protein kinase II (CaMKII)-mediated hyperphosphorylation of ryanodine receptor 2 (RyR2), which could result in increased sarcoplasmic reticulum (SR) Ca^2+^ leak, delayed afterdepolarizations (DADs), and subsequent AF occurrence [12, 13]. In our experiments, atrial-specific knockdown (KD) of Zbtb16 or Txnip by adeno-associated virus 9 (AAV9) was found to result in reduced ROS generation and ameliorated AF susceptibility in db/db mice. Taken together, our results revealed that inhibiting Zbtb16 might be able to decrease AF risk through downregulating Txnip-Trx2 signaling, which provided a novel therapeutic target for AF in the setting of T2DM.

## Materials and methods

### Experimental animals

The BKS.Cg-*Dock7*^*m*^ + */* + *Lepr*^*db*^/J db/ + and db/db mice were purchased from the Jackson Laboratory (Stock number: 000642, Bar Harbor, ME). The male and female mice at 8–10 weeks of age received 100 μL of AAV9 (1.0 × 10^12^ vector genomes/mL) in PBS via tail vein injection and sacrificed 4 weeks later. Atrial-specific gene delivery was constructed based on AAV9 vector according to previous report [14]. AAV9-scramble negative control (AAV9-NC), AAV9-Zbtb16 specific short hairpin RNA (AAV9-shZbtb16), and AAV9-Txnip specific short hairpin RNA (AAV9-shTxnip) were purchased from Hanbio Biotech Co., Ltd. (Shanghai, China). The db/ + and db/db mice at 8–10 weeks of age received intraperitoneal injection of Mito-TEMPO (S9733; Selleck Chemicals, Houston, TX, USA) (0.7 mg/kg per day) or vehicle for 4 weeks to observe ROS generation changes [15].

### Mouse model of AF

AF model mice were established by injection with acetylcholine (ACh, S1805; Selleck Chemicals, Houston, TX, USA)-CaCl_2_ (66 μg/mL ACh and 10 mg/mL CaCl_2_) daily via the tail vein at 1 mL/kg for 7 days. Control mice were injected with 0.9% of normal saline daily via the tail vein at 1 mL/kg for 7 days [16].

### Intracardiac programmed electrical stimulation

The mouse electrocardiogram (ECG) at baseline and during intracardiac programmed electrical stimulation were recorded as described previously [17]. Briefly, a 1.1-F electrophysiological catheter (ERP-800, Millar Inc, Houston, TX) was inserted into right atrium via the right jugular vein of mouse. Three trains of 2 s burst pacing were performed: the first 2 s burst was applied at a cycle length of 40 ms with a pulse duration of 5 ms. After 3 min of stabilization, the second 2 s burst was applied at a cycle length 20 ms with a pulse duration of 5 ms. Following another 3 min of stabilization, the last 2 s burst was applied at a cycle length of 20 ms with a pulse duration of 10 ms. AF was defined as rapid, irregular atrial response of > 1 s [18].

### RNA sequencing

Total RNA was isolated from left atria of db/db mice and db/ + littermates (*n* = 3 in each group) using mirVana miRNA Isolation Kit (AM1561; Thermo Fisher Scientific, Waltham, MA). NanoDrop 2000 spectrophotometer (Thermo Fisher Scientific, Waltham, MA) and RNA Nano 6000 Assay Kit of the Agilent Bioanalyzer 2100 system (Agilent Technologies, CA, USA) were used to evaluate the RNA purity, concentration, and integrity. The libraries were generated using NEBNext Ultra™ RNA Library Prep Kit for Illumina (E7770, NEB, USA) following manufacturer’s recommendations and index codes were added to attribute sequences to each sample. Low-quality reads of the raw data were abandoned and all the analyses were based on high-quality clean data. Results with fold change > 2 or < 0.5 and adjusted P < 0.05 were assigned as differentially expressed genes (DEGs), and Kyoto Encyclopedia of Genes and Genomes (KEGG) pathway enrichment and Gene Ontology (GO) enrichment analysis were performed subsequently.

### CUT&Tag assay

Cells were harvested from left atria of db/db mice and db/ + littermates. For each prepared sample, concanavalin-A-coated magnetic beads were added and incubated at room temperature for 15 min. The bead-bound cells were resuspended, treated with the Zbtb16 primary antibody (1:50; sc-28319; Santa Cruz, USA) and placed on a rotating platform overnight at 4 °C. Next, cells were incubated in the presence of the secondary antibody at room temperature for 30 min. Then cells were incubated with the pA-Tn5 adapter complex at room temperature for 1 h, resuspended in Tagmentation Buffer, and incubated at 37 °C for 1 h. Then proteinase K digestion and bead purification were performed for DNA extraction. GenSeq® 2 × HiFi PCR Mix (GenSeq Inc.) was used to amplify CUT&Tag libraries. Next, the libraries were qualified using Agilent 2100 bioanalyzer and sequenced in a NovaSeq platform (Illumina) with GenSeq® CUT&Tag kit (GenSeq Inc.) subsequently. The CUT&Tag assay analysis was finished with the technical support of CloudSeq Biotech (Shanghai, China).

### Histological analysis and immunofluorescence

Mouse hearts were fixed with 4% paraformaldehyde, embedded in paraffin and sectioned at 5 μm thickness. Then Sirius red staining was performed. For immunofluorescence staining, mouse heart sections were stained with primary antibodies against Flag (8146S; Cell Signaling Technology, Danvers, MA), α-Actinin (3134S; Cell Signaling Technology, Danvers, MA), COX IV (11242-1-AP; Proteintech Group, Inc, Chicago, IL) or Txnip (A9342; ABclonal, Wuhan, China). Nuclei were stained with DAPI (C1002; Beyotime, Shanghai, China). All images were captured with an Olympus microscope (Olympus, Japan) or CaseViewer (3DHISTECH Ltd, Budapest, Hungary) and measured by Image J software (National Institute of Health, Bethesda, Maryland, USA).

### Atrial myocytes isolation

Atrial myocytes were isolated from adult mice as previously described [19]. In, brief, db/db mice and db/ + littermates were heparinized (100 U/kg, i.p.) and euthanized with pentobarbital sodium (50 mg/kg, i.p.). The hearts were rapidly removed and mounted to a Langendorff apparatus. Ca^2+^-free Tyrode’s solution (in mM: 135 NaCl, 5.4 KCl, 1 MgCl_2_, 0.33 NaH_2_PO_4_, 10 HEPES, 5 Taurine, 10 2–3-butanedione monoxime and 10 glucose, pH 7.4 (NaOH)) was used to perfuse the hearts through the aorta (37 °C, 3 mL/min) for 5 min and the same solution containing collagenase type II (1 mg/mL, 17,101,015; Thermo Fisher Scientific Inc, Waltham, MA) was perfused for 20 min. Then the atrium was dissected and placed in a tube containing KB solution (in mM: 85 KOH, 50 K-glutamate, 30 KH_2_PO_4_, 20 taurine, 30 KCl, 1.0 MgCl_2_, 0.5 EGTA, 10 HEPES, and 10 glucose, pH 7.4 (KOH)), single cell was obtained with gently mechanical agitating.

### Patch-clamp recording

All experiments were performed at 37 ℃. Atrial myocytes were maintained in Tyrode’s solution (in mM: 135 NaCl, 5.4 KCl, 1 MgCl_2_, 0.33 NaH_2_PO_4_, 10 HEPES, 1.8 CaCl_2_, 5 Taurine, 10 2-3-butanedione monoxime and 10 glucose, pH 7.4 (NaOH)) for 5 min. For electrophysiological recordings, patch pipettes (resistance 2–4 MΩ) were filled with pipette solution (in mM: 110K-aspartate, 30 KCl, 5 NaCl, 10 HEPES, 0.1 EGTA, 5 MgATP, 5 creatine phosphate, and 0.05 CAMP, pH 7.2 (KOH)). Data were measured using MultiClamp 700B patch-clamp amplifier (Axon Instruments, Sunnyvale, CA, USA) and recorded by pCLAMP 10 software (Molecular Devices, Sunnyvale, CA, USA) as described previously [20]. Sequential electric stimuli (1 Hz) were used to induce action potentials (APs). Another 30 s was followed to observe the delayed afterdepolarization (DAD, defined as > 1 mV depolarization within 0.5 s) [12, 21].

### Intracellular Ca^2+^ imaging

Spontaneous Ca^2+^ waves were imaged at 37 ℃ with the Leica SP8 STED inverted microscope (Leica, Germany) fitted with a 63 × oil immersion objective. As described previously, intact atrial myocytes were maintained in Tyrode’s solution contained 15 μM Fluo-4 AM for 20 min (40704ES50; Yeasen, Shanghai, China) and the scan-line was placed across the length of the cell in a medial plane (1.67 ms/line, 3000 lines) for recording [22, 23]. Calcium transients were recorded by the EMCCD camera with the 480 ± 20 nm excitation light and Fluo-4 AM fluorescence intensity was acquired at a sampling frequency of 120 Hz. After recording calcium transients at 1 Hz stimulation, 20 mM caffeine stimulation was performed to estimate the calcium content in the sarcoplasmic reticulum (SR).

### Measurement of ROS levels

Intracellular ROS was measured using dihydroethidium (DHE, 50102ES02; Yeasen, Shanghai, China). Cells were incubated with 10 μM DHE for 60 min at 37 °C protected from light and the fluorescence (518 nm excitation; 610 nm emission) was captured under a fluorescence microscope (Olympus, Japan) microscope.

### HL-1 cell culture, adenovirus (AD) transfection, and Mito-TEMPO treatment

Mouse atrial myocyte-derived cell line HL-1 was maintained in complete Claycomb medium (51800C; Sigma-Aldrich, St. Louis, MO) containing 10% fetal bovine serum, 100 U/mL penicillin/streptomycin, 2 mmol/L L-glutamine, and 100 μmol/L norepinephrine and incubated at 37 °C with 5% CO_2_ for 48 h. D-glucose (high glucose (HG), 30 mM) or d-mannitol (normal glucose (NG), 30 mM, as the osmotic control) was used for high glucose experiments as described previously [24]. The Zbtb16 knockdown adenovirus (AD) and Txnip knockdown AD were provided by Hanbio Biotech Co., Ltd. (Shanghai, China). HL-1 cells were infected with AD that added in 1 mL Claycomb medium containing 10% FBS in a 3.5 cm dish for 4–6 h, then Claycomb medium (1 mL) without AD was added in the culture dish. Eight hours later, the medium was replaced with 2 mL fresh Claycomb medium containing 10% FBS and cultured for another 24 h. Mito-TEMPO (10 μM, S9733; Selleck Chemicals, Houston, TX) or vehicle was added into the culture medium to evaluate ROS generation [25].

### Co-immunoprecipitation (IP) assay

Cells or tissues were lysed by IP lysis buffer (87787; Thermo Fisher Scientific, Waltham, MA) with protease inhibitor cocktail (B14001, Bimake, Houston, TX, USA). Protein A/G magnetic beads (B23202; Bimake, Houston, TX, USA) were used for co-IP assay following manufacturer’s recommendations. In brief, 50 μL magnetic beads were transferred to a 1.5 mL tube, then the magnetic beads were washed in binding buffer (50 mM Tris, 150 mM NaCl, 0.1%–0.5% Tween 20, pH 7.5). Whole-cell lysates were mixed with Txnip (14715S; Cell Signaling Technology, Danvers, MA), Trx2 antibody (A4424; ABclonal, Wuhan, China) (20 μg/mL) or IgG antibody (ab172730; Abcam, Cambridge, UK) for 1 h at room temperature. The beads were washed three times with the washing buffer. Then the proteins were eluted by boiling in 1 × SDS for 5 min at 95 ℃ for immunoblotting analysis.

### Protein extraction and Western blot analysis

Protein was extracted from HL-1 cells and mouse atrium tissues by RIPA lysis buffer (P0013B; Beyotime, Shanghai, China) containing a protease inhibitor cocktail (B14001, Bimake, Houston, TX, USA). Mitochondria Isolation Kit for Cell and Tissue cells (20128ES50; Yeasen, Shanghai, China) was used for mitochondria extraction. Protein was loaded on SDS-PAGE and transferred to PVDF membranes. The membranes were blocked by 5% filtered non-fat milk dissolved in TBST for 1 h and then incubated with the primary antibodies overnight at 4 °C, which included total-CaMKIIδ (1:1000; GTX111401; GeneTex, USA), phospho-CaMKIIδ (1:1000; ab182647; Abcam), oxidized CaMKII (1:1000; GTX36254; GeneTex, USA), O-GlcNAc (1:1000; sc-59623; Santa Cruz, USA), Txnip (1:1000; 14715S; Cell Signaling Technology, Danvers, MA), Trx2 antibody (1:1000; A4424; ABclonal, Wuhan, China), Zbtb16 (1:1000; sc-28319; Santa Cruz, USA), RyR2 (1:1000; 19765-1-AP; Proteintech), Phospho-RyR2 (1:1000; A010-31AP; Badrilla), SERCA2a (1:1000; ab150435; Abcam), Phospholamban (1:1000; ab219626; Abcam), Phospho-Phospholamban (1:1000; AP0910; ABclonal), ACTB (1:1000; 4970S; Cell Signaling Technology, Danvers, MA), Lamin B1 (1:1000, 13435S, Cell Signaling Technology) and COX IV (1:10,000; 11242-1-AP; Proteintech Group, Inc, Chicago, IL). Then the membranes were incubated with secondary antibody for 1 h at room temperature, and signals were visualized and quantified by chemiluminescence (WBKLS0500; Millipore, Darmstadt, Germany) on a chemiluminescence detection system (Tanon, Shanghai, China).

### Trx2 activity analysis

Thioredoxin Fluorometric Activity Assay Kit (No. 500228, Cayman, USA) was used to detect Trx2 activity in mitochondrial lysate according to the manufacturer’s instructions. Fluorescence was measured by a Synergy H4 Reader (BioTek, Winooski, VT, USA) with excitation and emission wavelengths of 520 and 560 nm, respectively.

### Luciferase reporter assays and plasmid construction

Luciferase reporter assays were analyzed as previous described [26]. Briefly, 293T cells cultured on six-well plates at 70–80% confluence were co-transfected with pRL-TK plasmid, Zbtb16 plasmid, and pGL3-Basic plasmid encoding predicted promoter sequence of Txnip or negative control using Lipofectamine 2000 (11668019; Thermo Fisher Scientific, Waltham, MA). The dual-luciferase reporter assay (e1910; Promega, CA, USA) was used to detect luciferase activity according to the manufacturer’s instructions using a chemiluminescent detector (Centro XS^3^ LB960, Germany) and the results were normalized to Renilla luciferase activity.

### Real‑time PCR analysis

RNAiso Plus (9109; Takara, Kusatsu, Japan) was used to extract total RNA, the RNA purity and concentration were evaluated by NanoDrop 2000 spectrophotometer (Thermo Fisher Scientific, Waltham, MA). PrimeScript RT reagent kit (RR036A; Takara, Kusatsu, Japan) was used to synthesize cDNA. ChamQ Universal SYBR qPCR Master Mix (Q711; Vazyme, Nanjing, China) was used for quantitative real-time polymerase chain reaction (RT-PCR) on the QuantStudio 3 Real-Time PCR System (Applied Biosisytems, Waltham, MA). The sequences of primers are shown in Table S1.

### Statistical analysis

Data were acquired and analyzed by pCLAMP software (version 10.1, Molecular Devices, LLC, USA), Origin 7 software (Microcal Software, USA), GraphPad Prism software (version 8.0.2, GraphPad Software, La Jolla, USA), and SPSS (version 22.0, IBM, USA). Results are presented as means ± standard error of means (SEM) or percentage. Kolmogorov–Smirnov test or Shapiro–Wilk was used to test the normality of data distribution before parametric or non-parametric tests. Statistical significance was assessed using two-tailed Student’s *t* tests between two groups, one-way or two-way ANOVA analysis with Bonferroni post hoc analysis among multiple groups and Fisher’s exact test for percentage values. Statistical significance was defined as *P* < 0.05.

## Results

### The db/db mice displayed higher AF vulnerability and increased Zbtb16 expression in atria

**Fig. 1:**
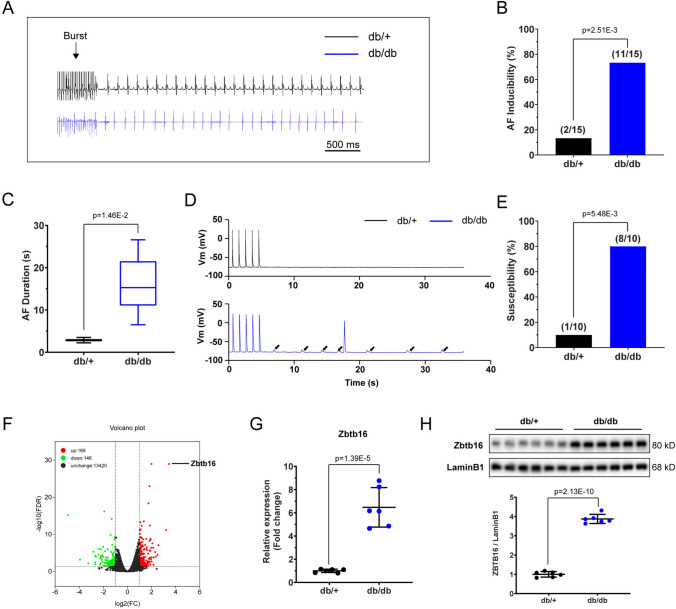
The db/db mice showed increased AF vulnerability with elevated Zbtb16 expression in atria. **A** Representative surface ECGs of db/ + and db/db mice induced by intracardiac electrical stimulation. Bar = 500 ms. **B**,** C** AF inducibility (**B**, *n* = 15 for each group) and duration (**C**) analysis. **D**, **E** Representative DAD images (**D**, solid arrow) of db/ + and db/db mouse atrial myocytes recorded by patch-clamp and analysis of susceptibility (**E**, *n* = 10 for each group). **F** The volcano plot of DEGs between db/ + and db/db mouse atria (*n* = 3 in each group). **G** RT-PCR analysis of Zbtb16 mRNA in db/ + and db/db mouse atria (*n* = 6 for each group). **H** Analysis of Zbtb16 protein expression in db/ + and db/db mouse atria (*n* = 6 for each group). Data in **B**, **E** were analyzed by Fisher’s exact test. Data in **C, G** and **H** were analyzed by two-tailed student *t* test. AF, atrial fibrillation

The db/db mice showed increased body mass and hyperglycemia as reported [27] (Fig. S1A, B). Intracardiac programmed electrical stimulation was performed in db/db mice and db/ + littermates (Fig. 1A), and db/db mice presented elevated AF inducibility (73.33% vs. 13.33%, *P* < 0.05) (Fig. 1B) and duration (15.87 ± 1.85 s vs. 2.86 ± 0.64 s, *P* < 0.05) (Fig. 1C). Patch-clamp recording of atrial myocyte evidenced more occurrence of arrhythmogenic DADs in atrial myocytes of db/db mice than in atrial myocytes of db/ + littermates during current-clamp simulation (80.00% vs. 10.00%, *P* < 0.05) (Fig. 1D, E). To investigate the key gene contributed to higher AF vulnerability in db/db mice, RNA-seq was performed on left atria of db/db mice and db/ + littermates (*n* = 3 in each group, fold change > 2 or < 0.5 and adjusted *P* < 0.05 were defined as differentially expressed genes (DEGs)). There were 314 DEGs, among them, Zbtb16 was identified as the most significantly upregulated gene (Fig. 1F). Further RT-PCT and Western blot analysis verified increased mRNA and protein expression of Zbtb16 in left atrium tissues of db/db mice (Fig. 1G, H), which was consistent with the RNA-seq results. In addition, the expression of Zbtb16 in atria and ventricles was compared by Western blot and the results suggested that Zbtb16 was mainly located in atria (Fig. S2A, B). These data indicated that increased AF vulnerability in db/db mice might be related to upregulated Zbtb16 expression.

### Txnip was regulated by Zbtb16 and combined with Trx2 in mitochondria of atrial myocytes

**Fig. 2:**
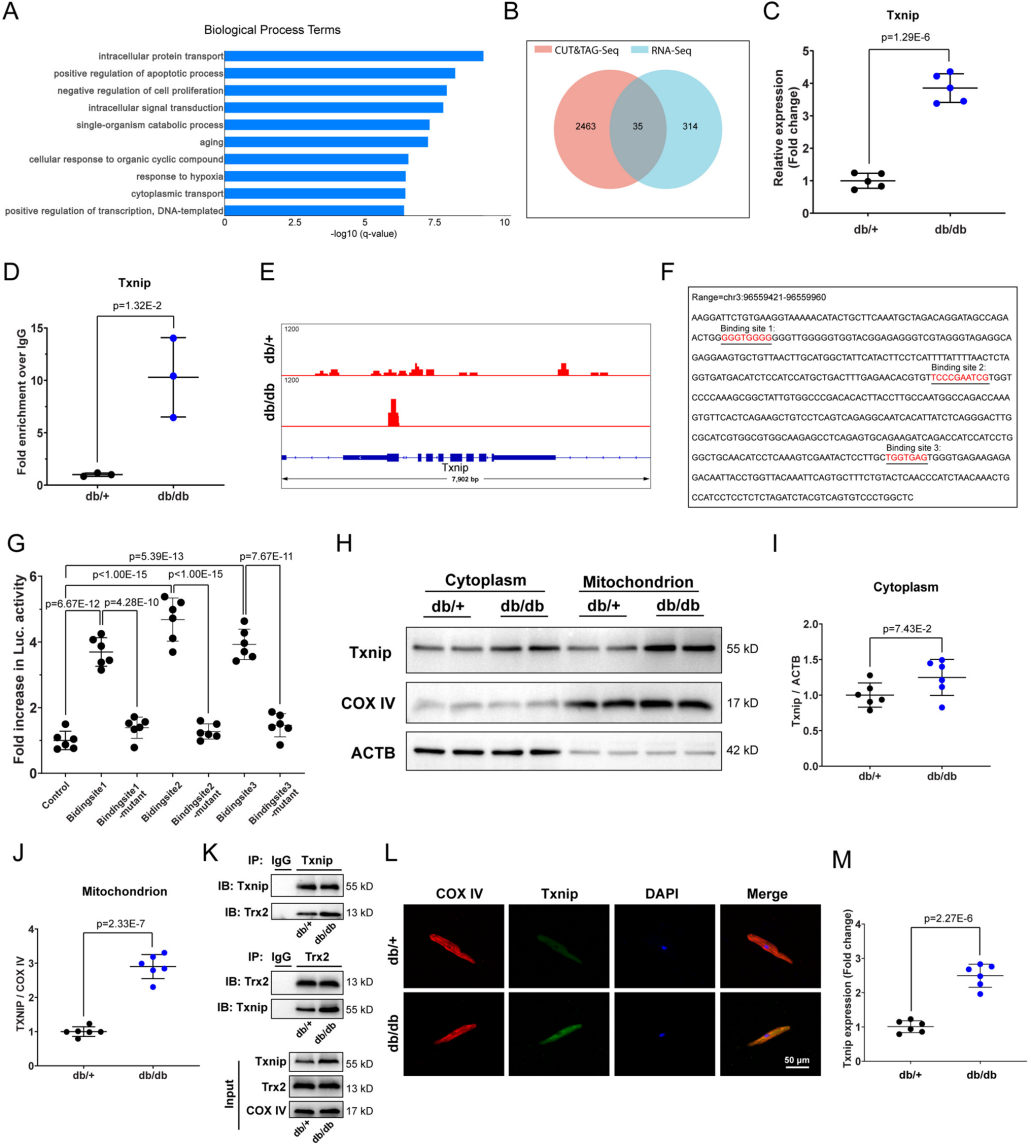
Txnip was regulated by Zbtb16 and combined with Trx2 in mitochondria. **A** Top ten enriched biological process terms by GO analysis in db/ + and db/db mouse atria. **B** Intersection of RNA-seq and CUT&Tag assay results of db/ + and db/db mouse atria (*n* = 3 in each group). **C** RT-PCR analysis of Txnip mRNA in db/ + and db/db mouse atria (*n* = 5 for each group). **D** CUT&Tag-PCR analysis of Txnip promoter binding region (*n* = 3 in each group). **E** CUT&Tag IGV visual analysis of Zbtb16 binding peak at the Txnip promoter region in db/ + and db/db mouse atria. **F**, **G** Binding site sequences of predicted Txnip promoter region (**F**) and luciferase reporter assay confirmation (**G**). **H–J** The respective WB images of cytoplasm and mitochondria Txnip expression (**H**) in db/ + and db/db mouse atria, (**I**, **J)** is the quantitative analysis of (**H**) (*n* = 6 for each group). **K** co-IP assay of Txnip-Trx2 combination in mitochondrion (*n* = 6 for each group). **L**, **M** Immunofluorescence co-staining for COX IV with Txnip in db/ + and db/db mouse atrial myocytes (**L**), (**M**) is the quantitative analysis of (**L**) (*n* = 6 for each group). Bar = 50 μm. Data in (**C**, **D**, **I**, **J** and **M**) were analyzed by two-tailed student *t* test. Data in **G** were analyzed by one-way ANOVA with Bonferroni’s multiple comparisons test

To investigate the downstream genes regulated by Zbtb16, we first identified the top ten biological process term based on RNA-seq results and Gene Ontology (GO) database (Fig. 2A). Then we performed CUT&Tag assay on left atria of db/db mice and db/ + littermates to identify Zbtb16 target genes, which showed there were 35 intersected genes based on RNA-seq and CUT&Tag assay results (Fig. 2B). Among the intersection genes, only Txnip was included in the top one enriched biological process term of RNA-seq, which suggested Txnip could be the key gene regulated by Zbtb16 directly. The upregulation of Txnip gene was verified by RT-PCR (Fig. 2C) and Txnip promoter binding region of Zbtb16 was proved by CUT&Tag-PCR (Fig. 2D). The IGV visual analysis showed that there was a manifested Zbtb16 binding peak at the Txnip promoter region in atria of db/db mice but not in the atria of db/ + littermates (Fig. 2E). In addition, the binding of Zbtb16 and Txnip promoter region was revealed for the first time in our study and there were no known binding site sequences from de novo motif search analysis. To identify the binding sites in Txnip promoter region, we truncated this region (chr3:96559421–96559960) predicted by CUT&Tag and verified three binding sites which were further confirmed by luciferase reporter assay (Fig. 2F, G). In cardiomyocyte, it was known that Txnip combined with Trx1 at cytoplasm or Trx2 at mitochondrion [8, 9]. Thus, Western blot was used to analyze the Txnip expression in atria of db/db mice and db/ + littermates, results showed Txnip was upregulated distinctly in mitochondria than the cytoplasm in atria of db/db mice (Fig. 2H–J). We further performed co-IP assay and results illustrated that the combination of Txnip-Trx2 was increased in atrium mitochondria of db/db mice compared to db/ + littermates (Fig. 2K). Moreover, immunofluorescent analysis indicated that Txnip co-localized with mitochondrial marker COX IV and the expression of Txnip is increased in atrial myocytes of db/db mouse (Fig. 2L, M). All these results revealed that Txnip expression was upregulated by Zbtb16 in atria of db/db mice and Txnip could combine with Trx2 in mitochondria.

### Zbtb16 or Txnip knockdown decreased Txnip-Trx2 combination and ameliorated Trx2 function in atria of db/db mice

**Fig. 3:**
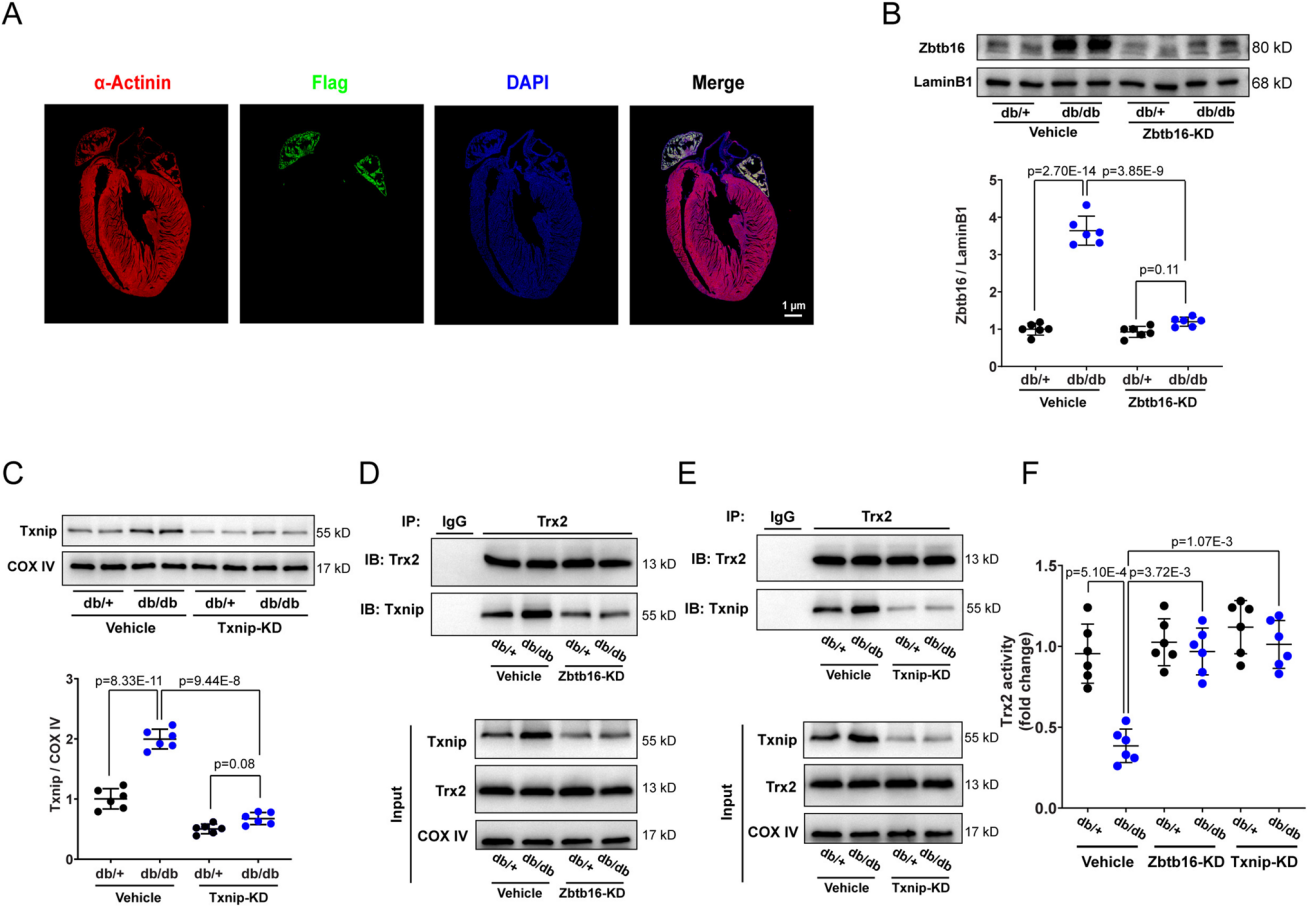
Zbtb16 or Txnip knockdown reduced combination of Txnip-Trx2 and improved Trx2 function. **A** Immunofluorescence co-staining for α-Actinin with Flag in mouse heart. **B**, **C** The respective WB images and quantitative analysis of Zbtb16 expression (**B**) in db/ + and db/db mouse atrial myocytes and Txnip expression (**C**) in mitochondria (*n* = 6 for each group). **D**, **E** co-IP assay of Txnip-Trx2 combination in mitochondria after Zbtb16-KD (**D**) or Txnip-KD (**E**) (*n* = 6 for each group). **F** Analysis of Trx2 activity in mitochondria of db/ + and db/db mouse atria (*n* = 6 for each group). Bar = 1 μm. Data in (**B, C**, and **F**) were analyzed by two-way ANOVA with Bonferroni’s multiple comparisons test. *KD* knockdown

To investigate the function of Zbtb16 and Txnip in atrial myocytes, we constructed atrial-specific gene delivery based on AAV9 vector with the Flag tag [14]. Immunofluorescent stain showed the Flag protein mainly expressed in mouse atria (Fig. 3A) after tail vein injection of the AAV9. Then we transfected the atrial-specific Zbtb16-KD AAV9 or Txnip-KD AAV9 via tail vein injection in db/db mice and db/ + littermates, Western blot results showed the significant decrease of Zbtb16 and Txnip expression in atria (Fig. 3B, C). Moreover, co-IP analysis revealed that Zbtb16 or Txnip knockdown decreased Txnip-Trx2 combination in db/db mouse atrium mitochondria (Fig. 3D, E). As reported, Txnip combined with Trx2 could inhibit the activity of Trx2 [7]; accordingly, our results verified that Trx2 activity was decreased in atrium mitochondria of db/db mice and could be reversed by Zbtb16 or Txnip knockdown (Fig. 3F). These findings suggested that the Zbtb16 or Txnip knockdown decreased the Txnip-Trx2 combination and ameliorated Trx2 function in atrial myocyte mitochondria.

### Zbtb16 or Txnip knockdown decreased generation of ROS and activation of CaMKIIδ in atria of db/db mice

**Fig. 4:**
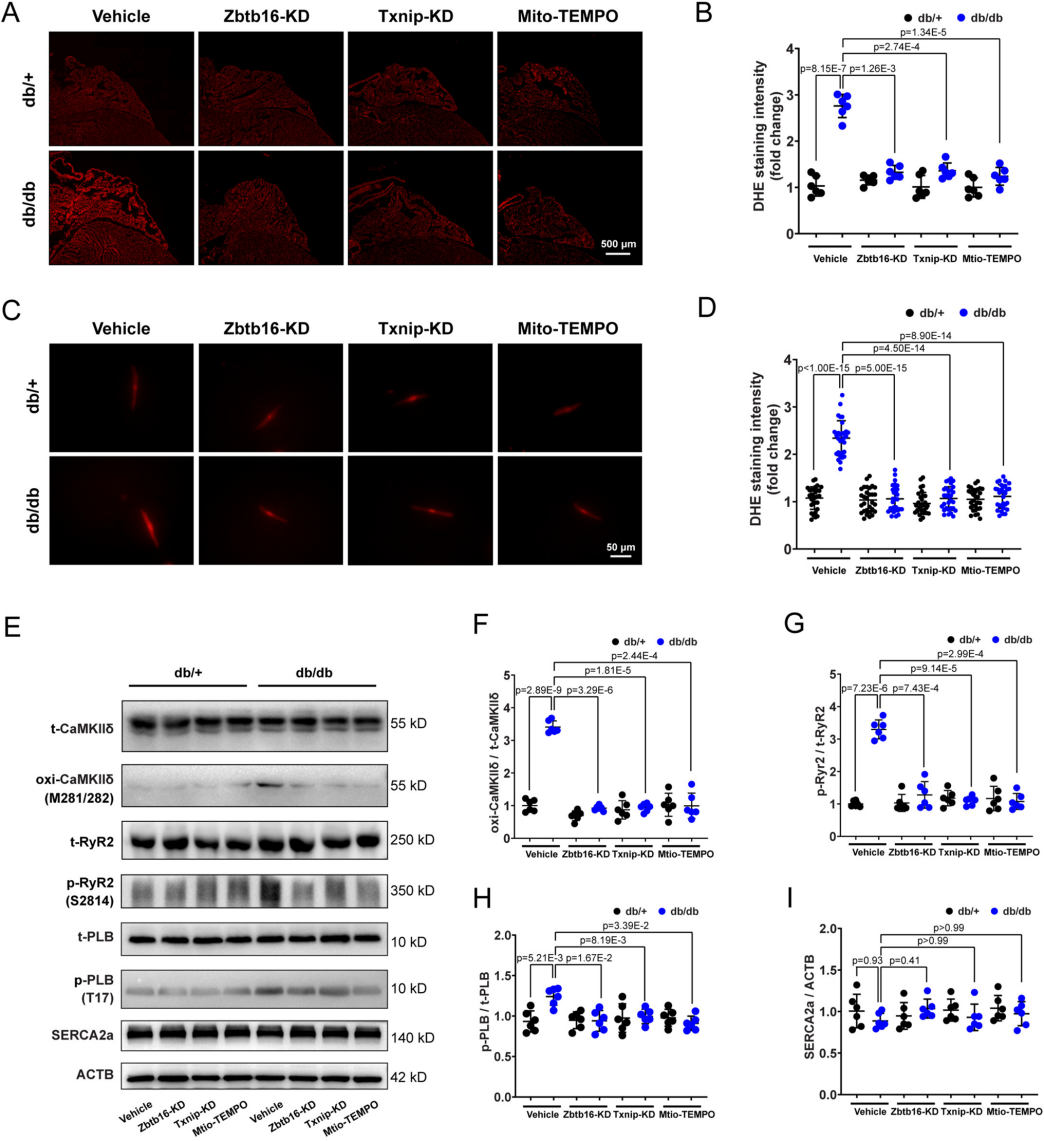
Zbtb16 or Txnip knockdown reduced ROS generation and CaMKIIδ activation.** A**, **B** Representative images (**A**) and quantitative analysis (**B**) of ROS level measured by DHE staining in db/ + and db/db mouse atria with Zbtb16-KD, Txnip-KD or Mito-TEMPO treatment (n = 6 for each group). Bar = 500 μm. **C**, **D** Representative images (**C**) and quantitative analysis (**D**) of ROS level measured by DHE staining in db/ + and db/db mouse atrial myocytes (*n* = 30 from 6 mice, 5 cells were calculated in each mouse). Bar = 50 μm. **E**–**I** Representative WB images (**E**) and quantitative analysis in db/ + and db/db mouse atria of CaMKIIδ (**F**), RyR2 (**G**), PLB (**H**) and SERCA2a (**I**) with Zbtb16-KD, Txnip-KD or Mito-TEMPO treatment (*n* = 6 for each group). Data in (**B**, **D**, and **F**–**I**) were analyzed by two-way ANOVA with Bonferroni’s multiple comparisons test. *KD* knockdown

As Trx2 is involved in the mitochondrial ROS generation [7], we performed DHE staining to measure the level of ROS in atria of db/db mice and db/ + littermates. The results displayed increased ROS production in atria of db/db mice, and this could be reversed by Zbtb16-KD, Txnip-KD or Mito-TEMPO treatment (Fig. 4A, B). We further isolated atrial myocytes from db/ + and db/db mice to measure the level of ROS, the results were consistent with the atrial tissue (Fig. 4C, D). Excess ROS generation is closely related to DADs occurrence and AF initiation, the oxidation and activation of CaMKII was known to play an important role in this process [12]. Thus, Western blot was used to analyze the expression of total-CaMKIIδ (t-CaMKIIδ) and oxidized-CaMKIIδ (oxi-CaMKIIδ) in atrial tissues. The elevated expression of oxi-CaMKIIδ was displayed in atria of db/db mice and could be inhibited by Zbtb16-KD, Txnip-KD or Mito-TEMPO treatment (Fig. 4E, F). Moreover, the phosphorylated-CaMKIIδ (p-CaMKIIδ) and O-GlcNAc expression differences showed no significance after Zbtb16 or Txnip knockdown (Fig. S3A-D). Since the ryanodine receptor 2 (RyR2) and phospholamban (PLB) can be activated by CaMKIIδ and work with SERCA2a in maintaining SR Ca^2+^ content [28], Western blot analysis was performed and results showed that phosphorylated-RyR2 (p-RyR2) expression was increased manifestly in atria of db/db mice (Fig. 4G). At the same time, the phosphorylated-PLB (p-PLB) expression was slightly increased (Fig. 4H). After Zbtb16-KD, Txnip-KD or Mito-TEMPO treatment, the upregulation of p-RyR2 and p-PLB was decreased. In addition, the expression of SERCA2a was similar in atria between db/db group and db/ + littermates with or without these treatments (Fig. 4I). Taken together, these findings indicated that Zbtb16-Txnip-Trx2 signaling contributed to increased ROS generation and CaMKIIδ activation in atria of db/db mice.

### Zbtb16 or Txnip knockdown attenuated spontaneous Ca^2+^ waves (SCWs) and reverted SR Ca^2+^ content in atrial myocytes of db/db mouse

**Fig. 5:**
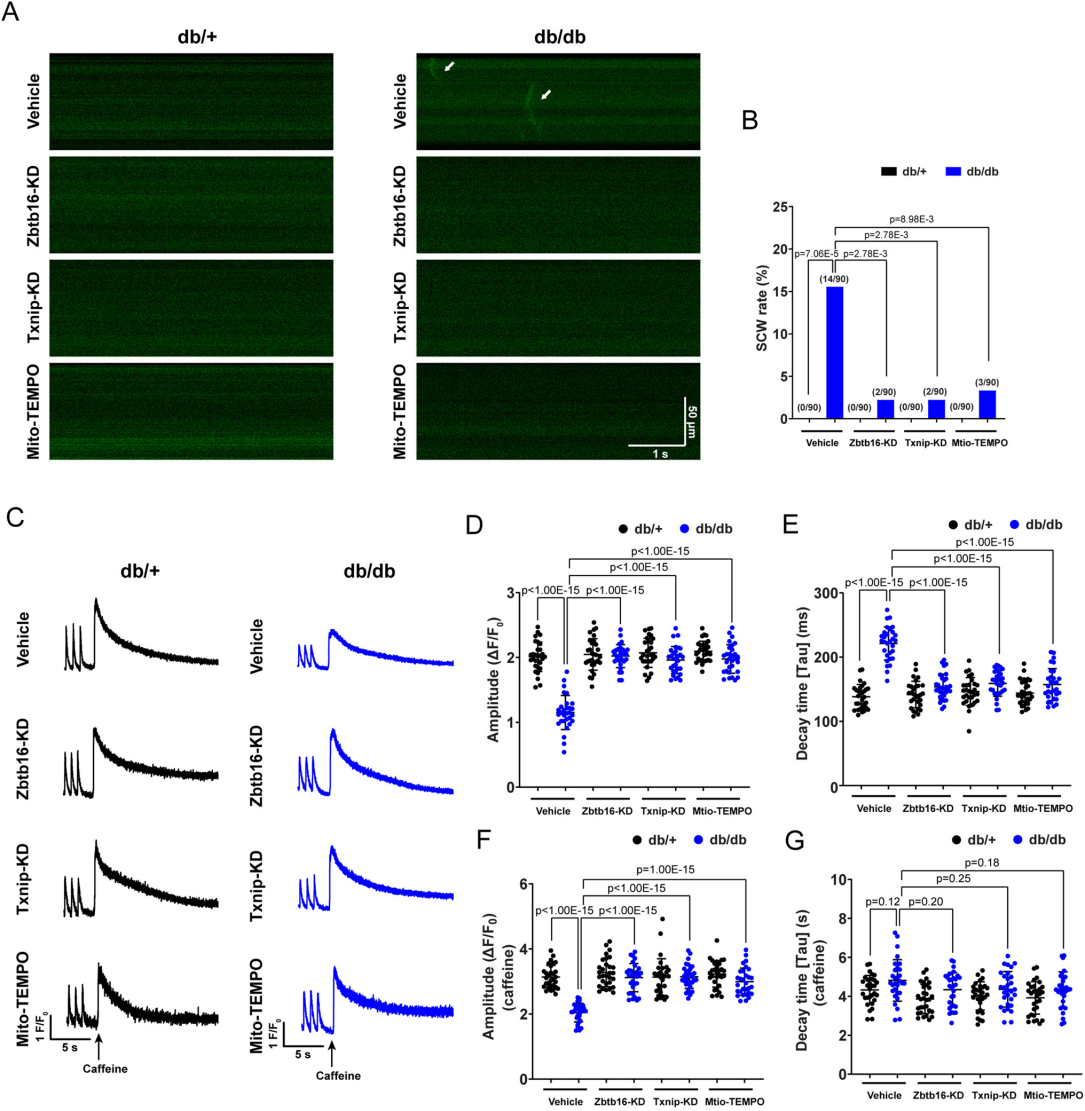
Zbtb16 or Txnip knockdown decreased SCWs and recovered SR Ca^2+^ content. **A**, **B** Representative confocal line-scan Ca^2+^ images by Fluo-4 AM Ca^2+^ indicator staining (**A**, solid arrow) and SCWs analysis of atrial myocytes (**B**, *n* = 90 from 6 mice, 15 cells were observed in each mouse). Bar = 50 μm, 1 s. **C** Representative traces of Ca^2+^ transients and caffeine-induced SR Ca^2+^ release in db/ + and db/db mouse atrial myocytes. Bar = 5 s. **D**, **E** The quantitative analysis of Ca^2+^ transient amplitude (**D**) and decay time τ (**E**). **F**, **G** The quantitative analysis of caffeine-induced SR Ca^2+^ release (**F**) and decay time τ (**G**) (*n* = 30 from 6 mice, 5 cells were calculated in each mouse). Data in **B** were analyzed by Fisher’s exact test. Data in **D**–**G** were analyzed by two-way ANOVA with Bonferroni’s multiple comparisons test. SCW; spontaneous Ca^2+^ wave. SR: sarcoplasmic reticulum

Abnormal SR Ca^2+^ release and uptake could affect the Ca^2+^ homeostasis and trigger arrhythmia. Thus, we isolated db/ + and db/db mouse atrial myocytes and performed the line-scan confocal to estimate abnormal Ca^2+^ release by Fluo-4 AM staining. The results revealed elevated occurrence of SCWs in db/db group, which could be inhibited by Zbtb16-KD, Txnip-KD or Mito-TEMPO treatment (Fig. 5A, B). We further measured Ca^2+^ transients and caffeine-induced Ca^2+^ release in mouse atrial myocytes. Compared with db/ + littermates, the amplitude of Ca^2+^ transients was decreased and the decay time τ was prolonged in atrial myocytes of db/db mouse, which could be reverted by Zbtb16-KD, Txnip-KD or Mito-TEMPO treatment (Fig. 5C–E). Moreover, the caffeine-induced Ca^2+^ release was reduced in db/db mouse atrial myocytes compared to db/ + littermates and Zbtb16-KD, Txnip-KD or Mito-TEMPO treatment could recover the SR Ca^2+^ content (Fig. 5F). Besides, although the decay time τ of caffeine-induced Ca^2+^ release showed trends toward upregulation in the db/db mouse atrial myocytes, there were no significant differences among groups with different treatments (Fig. 5G). These findings suggested that the Zbtb16 or Txnip knockdown improved the Ca^2+^ homeostasis in atrial myocytes of db/db mouse.

### Zbtb16 or Txnip knockdown ameliorated activity of Trx2 and attenuated ROS-induced CaMKIIδ activation in HL-1 cells

**Fig. 6:**
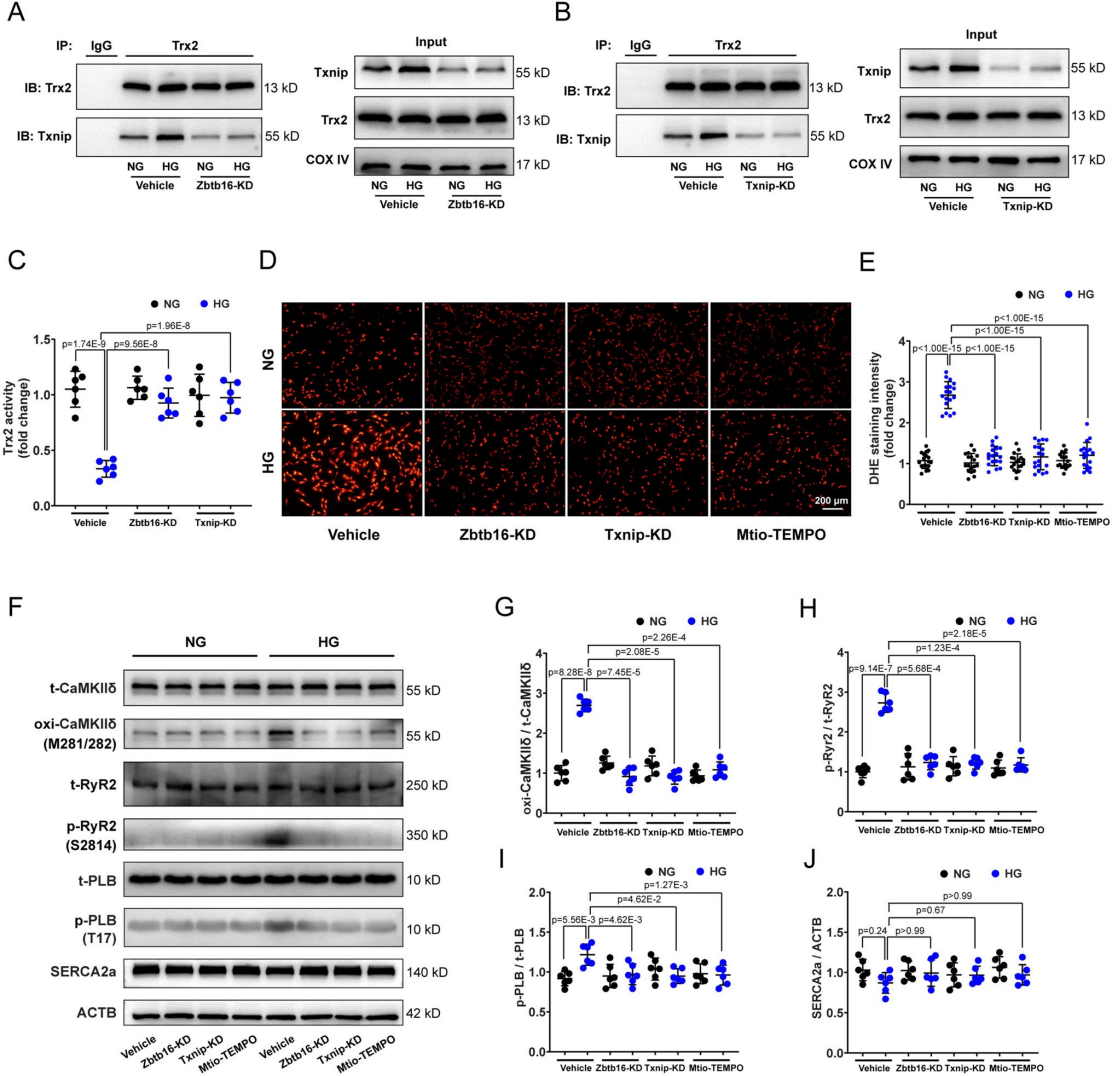
Zbtb16 or Txnip knockdown ameliorated Trx2 activity and reduced ROS-induced CaMKIIδ activation in HL-1 cells.** A**, **B** co-IP assay of Txnip-Trx2 combination in mitochondria after Zbtb16-KD (**A**) or Txnip-KD (**B**) in HL-1 cells (*n* = 6 for each group). **C** Analysis of Trx2 activity in mitochondria of HL-1 cells (*n* = 6 for each group). **D**, **E** Representative images (**D**) and quantitative analysis (**E**) of ROS level measured by DHE staining in HL-1 cells (*n* = 18). Bar = 200 μm. **F**–**J** Representative WB images (**F**) and quantitative analysis in HL-1 cells of CaMKIIδ (**G**), RyR2 (**H**), PLB (**I**), and SERCA2a (**J**) with Zbtb16- KD, Txnip-KD or Mito-TEMPO treatment (*n* = 6 for each group). Data in (**C**, **E** and **G**–**J**) were analyzed by two-way ANOVA with Bonferroni’s multiple comparisons test. *KD* knockdown

HL-1 cells, the mouse atrial cardiomyocyte line, was cultured in high glucose (HG, 30 mM) to mimic the diabetic condition in vitro. Co-IP assay results revealed that Zbtb16 or Txnip knockdown by adenovirus (AD) induced decreased combination of Txnip-Trx2 in HL-1 cells under HG condition compared to normal glucose (NG) group (Fig. 6A, B). At the same time, the activity of Trx2 was decreased in mitochondria of HL-1 cells under HG condition, which was reversed by Zbtb16 or Txnip knockdown (Fig. 6C). Besides, Zbtb16-KD, Txnip-KD or Mito-TEMPO treatment could reduce ROS generation (Fig. 6D, E) in HL-1 cells under HG condition. In line with the findings of mouse atria in vivo, the expression of oxi-CaMKIIδ and p-RyR2 was elevated manifestly in HL-1 cells under HG condition. However, p-PLB just increased slightly. Moreover, the elevated expression of oxi-CaMKIIδ, p-RyR2, and p-PLB could be inhibited by Zbtb16-KD, Txnip-KD, or Mito-TEMPO treatment (Fig. 6F–I). The expression of SERCA2a was similar between HG group and NG group with or without these treatments (Fig. 6J). These data indicated that Zbtb16-Txnip-Trx2 signaling contributed to increased ROS generation and CaMKIIδ activation in HL-1 cells under HG condition.

### Zbtb16 or Txnip knockdown reduced atrial fibrillation susceptibility and DADs generation in db/db mice

**Fig. 7:**
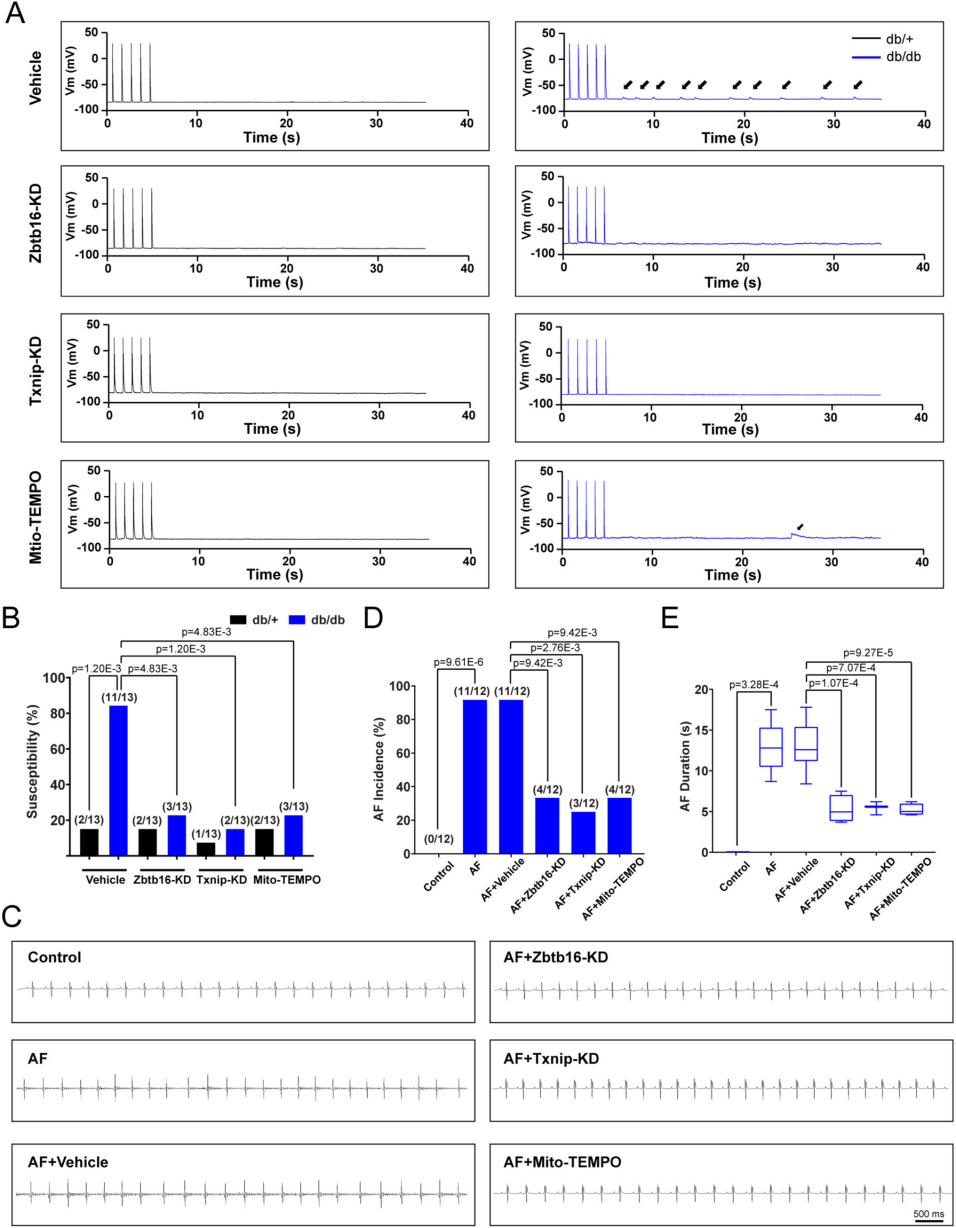
Zbtb16 or Txnip knockdown decreased susceptibility of atrial fibrillation and generation of DADs in db/db mice. **A**, **B** Representative DAD images (**A**, solid arrow) and quantitative analysis (**B**) of db/ + and db/db mouse atrial myocytes recorded by patch-clamp and analysis of susceptibility (*n* = 13 for each group). **C** Representative surface ECGs of ACh-CaCl_2_-induced AF model in db/db mice with Zbtb16-KD, Txnip-KD or Mito-TEMPO treatment. Bar = 500 ms. **D**, **E** AF incidence (**D**, *n* = 12 for each group) and duration (**E**) analysis. Data in **B**, **D** were analyzed by Fisher’s exact test. Data in **E** were analyzed by two-way ANOVA with Bonferroni’s multiple comparisons test. AF, atrial fibrillation, KD, knockdown

Finally, we utilized AAV9 to knockdown the expression of Zbtb16 or Txnip and performed Mito-TEMPO treatment in mice. As a consequence, the occurrence of DADs was reduced in db/db mice recorded by current-clamp simulation (Fig. 7A, B). Meanwhile, in the ACh-CaCl_2_-induced AF model of db/db mice, the incidence and duration of AF were decreased after Zbtb16-KD, Txnip-KD, or Mito-TEMPO treatment (Fig. 7C–E). Moreover, intracardiac programmed electrical stimulation was performed in db/db mice and db/ + littermates, results were consistent with the findings observed in ACh-CaCl_2_-induced AF model (Fig. S4A–C). Therefore, these data confirmed that Zbtb16 and Txnip were involved in the development of AF in db/db mice.

## Discussion

In this study, we demonstrated that the expression of Zbtb16 was increased in atria of db/db mice compared to the db/ + littermates. As a consequence, Zbtb16 upregulated Txnip transcription and much more Txnip combined with Trx2 in mitochondria, thus promoted excess ROS release, CaMKIIδ overactivation, plasma Ca^2+^ content increase, DADs occurrence, and increased AF susceptibility. Moreover, Zbtb16-KD, Txnip-KD or Mito-TEMPO treatment reduced incidence and duration of AF in ACh-CaCl_2_-induced AF model. Above results provided a new strategy for decreasing susceptibility of AF by targeting Zbtb16-Txnip-Trx2 pathway in T2DM.

Zbtb proteins are an evolutionary conserved family of transcriptional regulators and are characterized as containing C-terminal C2H2/Krüppel-type zinc finger domains and N-terminal BTB domain [29, 30]. Zbtb16, as the important member of Zbtb family, is a transcription factor that mainly regulates the development and function of innate-like unconventional T cells and is involved in the regulation of metabolism according to previous studies [31, 32]. However, the effects of Zbtb16 in the development of AF remain unknown. In this investigation, our results showed that Zbtb16 was increased significantly in the db/db mouse atria (Fig. 1F–H). The results showed that expression of Zbtb16 was elevated in db/db mice over time, displayed significance at 8 weeks (Fig. S2C, D). Due to Zbtb16 featured as transcription factor, we further performed CUT&Tag assay to search downstream signaling. Combined with the results of RNA-seq and CUT&Tag, we found that 35 genes existed in the intersection set and only Txnip was involved in the top 1 enriched biological process term of RNA-seq which manifested that Txnip might serve as the key downstream gene of Zbtb16 (Fig. 2A–C). Subsequently, we verified Zbtb16 regulated the expression of Txnip via binding with the promoter region for the first time by luciferase reporter assay (Fig. 2E–G). Trx is a thiol-disulfide oxidoreductase and plays a key role in the redox homeostasis [33]. Trx1 and Trx2 are localized primarily to the plasm and mitochondrion, respectively; however, Trx3 only exists in the testis [34]. Txnip could combine with Trx, which leads to the reduced function of against oxidative stress and decreased ROS scavenging. In our study, WB and co-IP results displayed increased Txnip mainly co-localized with Trx2 in mitochondria and resulted in much more ROS release in db/db mouse atria (Figs. 2H–M and 4A–D).

Mechanistically, mitochondria are involved in maintaining redox balance and excess mitochondrial ROS release has been implicated in the development of AF [35, 36]. Moreover, CaMKII, a ubiquitous and critical enzyme in cardiovascular system, contributed to activation of RyR2 and subsequent Ca^2+^ release from SR [37]. In cardiomyocytes, CaMKIIδ is the main isoform and could be activated via oxidative modification at Met281/282 induced by excess ROS generation [38]. Our results verified the increased expression of oxi-CaMKII and abnormal SR RyR2 Ca^2+^ release in db/db mouse atrial myocytes compared to db/ + littermates (Figs. 4E–G and 5A, B). Besides, the upregulated level of oxi-CaMKII and p-RyR2 was proved in HL-1 cells under HG condition, too (Fig. 6G, H). Because of sustaining activation of RyR2 in db/db mouse atrial myocytes, SR Ca^2+^ content and amplitude of Ca^2+^ transients were decreased (Fig. 5F). Popescu et al. reported that decreased amount of Ca^2+^ stored in the SR and the lower SR Ca^2+^ threshold led to the occurrence of Ca^2+^ waves and DADs in rat T2DM model. In this process, oxidative stress and activation of CaMKII and RyR2 played an important role [39]. In our study, after performing Zbtb16-KD, Txnip-KD or Mito-TEMPO treatment, the ROS generation and level of oxi-CaMKII reduced (Fig. 4A–F). As a result, SR Ca^2+^ content and amplitude of Ca^2+^ transients of db/db mouse atrial myocytes were reverted. In addition, SERCA2a plays a critical role for cytoplasm Ca^2+^ uptake to SR. Compared with db/ + littermates, results of Western blot revealed that the expression of SERCA2a showed no significant differences in db/db mouse atrial myocytes (Fig. 4I). But the Ca^2+^ transient decay time τ was prolonged, which represented the decreased activity of SERCA2a (Fig. 5E). This result might be due to the excess generation of ROS that could inhibit the activity of SERCA2a without significant changes of protein expression, which was verified by Balderas-Villalobos et al. [40]. Hence, when Zbtb16-KD, Txnip-KD or Mito-TEMPO treatment was performed, the generation of ROS was reduced and the Ca^2+^ transient decay time τ of db/db mouse atrial myocytes was recovered. Meanwhile, the Ca^2+^ uptake by SERCA2a was offset during caffeine stimulation, so the decay time τ of caffeine-induced Ca^2+^ transient showed no significant differences in db/db mouse atrial myocytes compared to db/ + littermates (Fig. 5G). PLB was involved in Ca^2+^ uptake regulation via SERCA2a [22]. Although the results showed increased expression of p-PLB in db/db mouse atrial myocytes, the caffeine-induced SR Ca^2+^ release still decreased significantly (Fig. 5F). This suggested that the slightly increased expression of p-PLB might be a compensation mechanism but failed to reverse the excess RyR2 Ca^2+^ leak activated by oxi-CaMKIIδ and the decreased SR Ca^2+^ content. As a result, susceptibility of DADs increased and AF would occur finally in db/db mouse atrial myocytes.

In addition, Erickson et al. demonstrated that O-GlcNAcylation modification of CaMKII was existed and spontaneous SR Ca^2+^ release was enhanced in diabetes [41]. However, Mesubi et al. reported that oxi-CaMKII and O-GlcNAcylation played critical but distinct roles in the mechanism for increased AF in diabetic mice, the RyR2 activated by oxi-CaMKII contributed to AF but the O-GlcNAcylation-induced AF was independent of CaMKII [11]. Thus, we detected the O-GlcNAcylation of atrium tissues from db/db mice and db/ + littermates, the results showed O-GlcNAcylation was unchanged after Zbtb16-KD or Txnip-KD which suggested O-GlcNAcylation was not involved in the Zbtb16-Txnip-Trx2 pathway (Fig. S3C, D). Besides, phosphorylation of CaMKIIδ (p-CaMKIIδ) also affected the SR Ca^2+^ release [39, 42], then we analyzed the expression of p-CaMKIIδ and there were no changes after Zbtb16-KD or Txnip-KD (Fig. S3A, B). These results revealed that Zbtb16-Txnip-Trx2 pathway mainly contributed to elevation of oxi-CaMKIIδ, increase of DADs, and occurrence of AF in db/db mice.

Moreover, targeting Zbtb16-Txnip-Trx2 signaling could reduce ROS release and represent cardioprotective effects clinically [43]. In cardiomyocytes, growing evidence suggested that Txnip-Trx2 system was a nodal point linking pathways of redox reaction by regulating ROS generation [44]. As reported previously, oxidative stress contributed to the atrial electrophysiological and structural remodeling in patients with AF and ROS-mediated abnormal SR Ca^2+^ homeostasis played a critical role in this process [45]. Purohit et al. demonstrated that diastolic SR Ca^2+^ leak and DADs were increased due to upregulated oxidation modification of CaMKII in the atrial tissue from patients with AF [46]. Overactivated oxi-CaMKII contributed to higher level phosphorylation of RyR2, reduced systolic Ca^2+^ transient amplitude, and decreased SR Ca^2+^ load, which was also observed in human induced pluripotent stem cells (hiPSCs) [38]. As a consequence, the abnormal Ca^2+^ handing resulted in the occurrence of Ca^2+^ waves, DADs, and AF. Besides, Chao et al. showed thiazolidinediones could prevent new-onset atrial fibrillation in patients with T2DM because of the potential antioxidant effects [47]. Lal et al. identified metformin as a candidate drug for AF treatment using hiPSC-derived atrial-like cardiomyocytes, and attenuated oxidative stress might be the underlying mechanism [48, 49]. Hence, targeting Zbtb16-Txnip-Trx2 pathway could serve as a novel therapeutic strategy for decreasing AF susceptibility in T2DM patients due to the important role in modulating ROS generation.

**Fig. 8:**
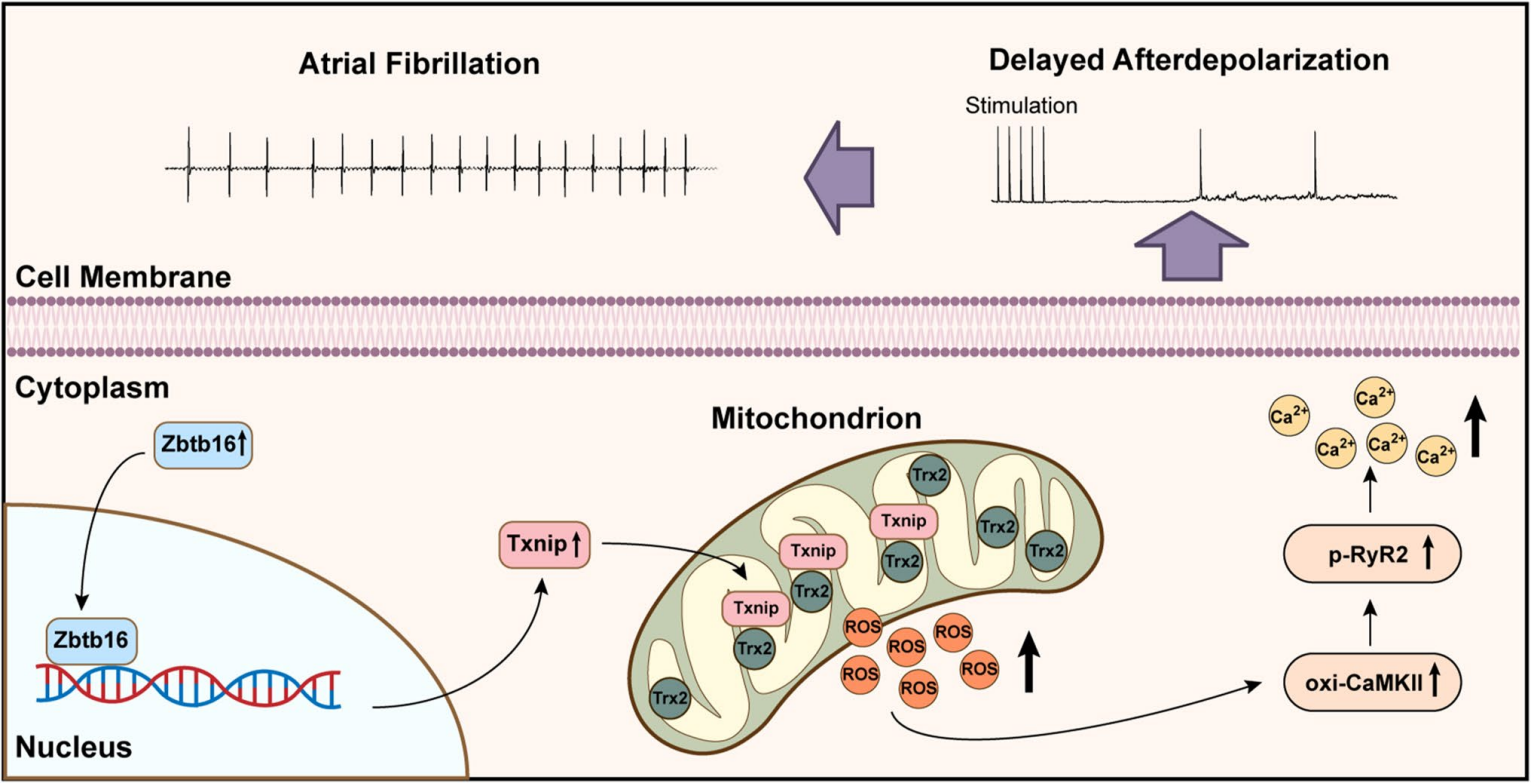
A working model of Zbtb16-induced DADs occurrence and AF development by Txnip-Trx2 signaling in db/db mice. Zbtb16-Txnip-Trx2 pathway contributed to AF development in db/db mice by excess ROS generation-induced CaMKIIδ overactivation, abnormal SR Ca^2+^ release and DADs occurrence

According to previous studies, atrial structural remodeling could provide a substrate for AF in obesity and T2DM mouse models [17, 50]. Liu et al. showed that the atrial structural remodeling including left atrial diameter expansion and interstitial fibrosis appeared at 12 weeks in db/db mice [51]. In our investigation, db/db mice were sacrificed at 12–14 weeks and displayed only slight increase in atrial interstitial fibrosis compared to db/ + littermates (Fig. S5A, B) which was consistent with previous reports [51]. However, ROS release was elevated manifested in atrial myocytes of db/db mouse at that time which was consistent with the high-level expression of Zbtb16 (Figs. 4A–D and S2C, D). Furthermore, Zbtb16-KD or Txnip-KD could inhibit the occurrence of DADs and reduce the incidence and duration of AF in ACh-CaCl2-induced AF model (Fig. 7A–E) with trends of atrial interstitial fibrosis area decreasing (Fig. S5B). These results revealed that DADs could occur and initiate AF generation due to excess ROS release and CaMKIIδ activation via Zbtb16-Txnip-Trx2 pathway (Fig. 8).

## Conclusion

We uncovered that Zbtb16-Txnip-Trx2 pathway might play an important role in AF development in db/db mice via the excess ROS generation and CaMKIIδ overactivation, abnormal SR Ca^2+^ release, and DADs occurrence. These findings provide experimental evidence for ameliorating AF development by interrupting Zbtb16-Txnip-Trx2 pathway.

## Study limitations

Several study limitations should be mentioned. First of all, atrial-specific Zbtb16 or Txnip knockout (KO) mice were not available in this study, so we performed atrial-specific gene delivery based on AAV9 vector to knockdown Zbtb16 and Txnip in db/db mice and db/ + littermates. Second, we used HL-1 cell line to perform experiments in vitro since there was no well-established protocol to culture adult mouse primary atrial myocytes. Although some studies provided probability for culturing [52, 53], atrial myocytes cultured in HG condition have not been proved and estimated.

### Supplementary Information

Below is the link to the electronic supplementary material.

Supplementary file1 (DOCX 6186 KB)

## Data Availability

All data used for this study are displayed or can be displayed upon request.

## Abbreviations

AF: Atrial fibrillation
DAD: Delayed afterdepolarization
T2DM: Type 2 diabetes mellitus
ROS: Reactive oxygen species
Zbtb16: Zinc finger and BTB (broad-complex, tram-track and bric-a-brac) domain containing 16
Txnip: Thioredoxin interacting protein
Trx2: Thioredoxin 2
CaMKII: Calcium/calmodulin-dependent protein kinase II
SR: Sarcoplasmic reticulum
RyR2: Ryanodine receptor 2
PLB: Phospholamban
HG: High glucose
NG: Normal glucose
SCW: Spontaneous Ca^2+^ wave
hiPSC: Human-induced pluripotent stem cell
