# Supplementary material for: Zbtb16 increases susceptibility of atrial fibrillation in type 2 diabetic mice via Txnip-Trx2 signaling
Source: Cell Mol Life Sci. 2024 Feb 13;81(1):88. doi: 10.1007/s00018-024-05125-2 (PMC10864461; doi:10.1007/s00018-024-05125-2)
Supplement: Supplementary file 1 — Supplementary file1 (DOCX 6186 KB) [file 18_2024_5125_MOESM1_ESM.docx]

**SUPPLEMENTARY** **MATERIAL**

**Zbtb16 increases susceptibility of atrial fibrillation in type 2 diabetic mice via Txnip-Trx2 signaling**

Zhi-Xing Wei^1^, Xing-Xing Cai^1^, Yu-Dong Fei^1^, Qian Wang^1^, Xiao-Liang Hu^1^, Cheng Li^1^, Jian-Wen Hou^2^, Yu-Li Yang^1^, Tai-Zhong Chen^1^, Xiao-Lei Xu^3^, Yue-Peng Wang^1^, Yi-Gang Li^1^.

^1^Department of Cardiology, Xinhua Hospital Affiliated to Shanghai Jiao Tong University School of Medicine, Shanghai, China. ^2^Department of Cardiology, The Second Affiliated Hospital of Zhejiang University School of Medicine, Hangzhou, Zhejiang Province, China. ^3^Department of Biochemistry and Molecular Biology, Department of Cardiovascular Medicine, Mayo Clinic, Rochester, United States.

Zhi-Xing Wei, Xing-Xing Cai and Yu-Dong Fei contribute equally to this study.

**Corresponding author:**

Yi-Gang Li, MD

Department of Cardiology, Xinhua Hospital Affiliated to Shanghai Jiao Tong University School of Medicine

Address: 1665 Kongjiang Road, Shanghai 200092, China

Tel: 86-21-2507-7260

Fax: 86-21-5596-4561

E-mail: [liyigang@xinhuamed.com.cn](mailto:liyigang@xinhuamed.com.cn)

**Table S1** Table of forward and reverse primers sequences for Real-Time PCR

| Gene | Forward Primer (5’ — 3’) | Reverse Primer (5’ — 3’) |
| --- | --- | --- |
| Zbtb16 | CTGCGGAAAACGGTTCCTG | GTGCCAGTATGGGTCTGTCT |
| Txnip | AGATACCCCAGAAGCTCCTCC | TGTCTTGAGAGTCGTCCACAT |
| LaminB1 | GAGTATGAGGCGGCACTAAAC | CATCTGCTAACTGCTTTTTGGC |
| COX IV | TCCCCACTTACGCTGATCG | GATGCGGTACAACTGAACTTTCT |

Zbtb16, zinc finger and BTB (broad-complex, tram-track and bric-a-brac) domain containing 16; Txnip, thioredoxin interacting protein


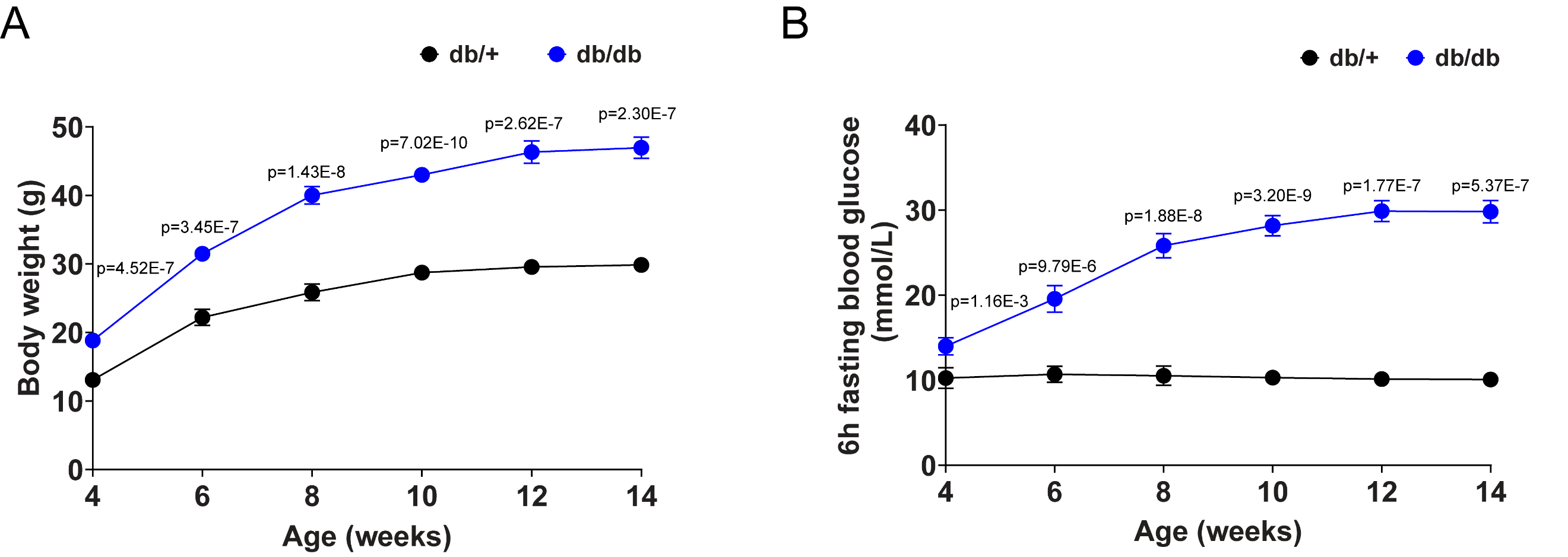


**Fig. S1** Body mass and blood glucose level of db/+ and db/db mice. **A** Body mass of db/+ and db/db mice (n=6). **B** Blood glucose level of db/+ and db/db mice (n=6). Data in (**A**, **B**) were analyzed by two-way ANOVA with Bonferroni’s multiple comparisons test


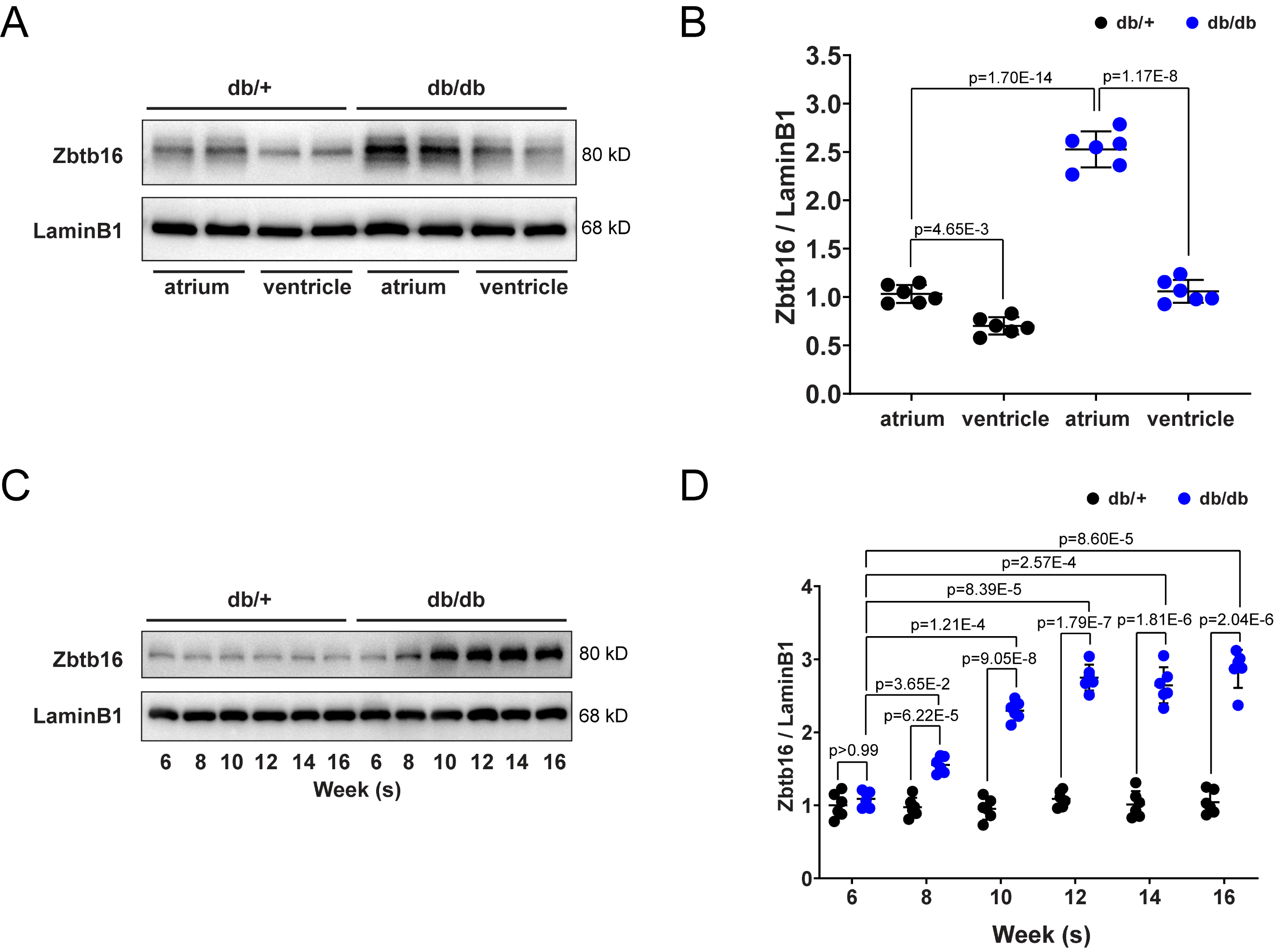


**Fig. S2** Zbtb16 expression in different heart component and time points of db/+ and db/db mice. **A**, **B** Representative Western blot images (**A**) and quantitative analysis of Zbtb16 expression (**B**) in db/+ and db/db mouse atria and ventricles (n=6). **C**, **D** Representative Western blot images (**C**) and quantitative analysis of Zbtb16 (**D**) in different age of weeks (n=6). Data in (**B**, **D**) were analyzed by two-way ANOVA with Bonferroni’s multiple comparisons test


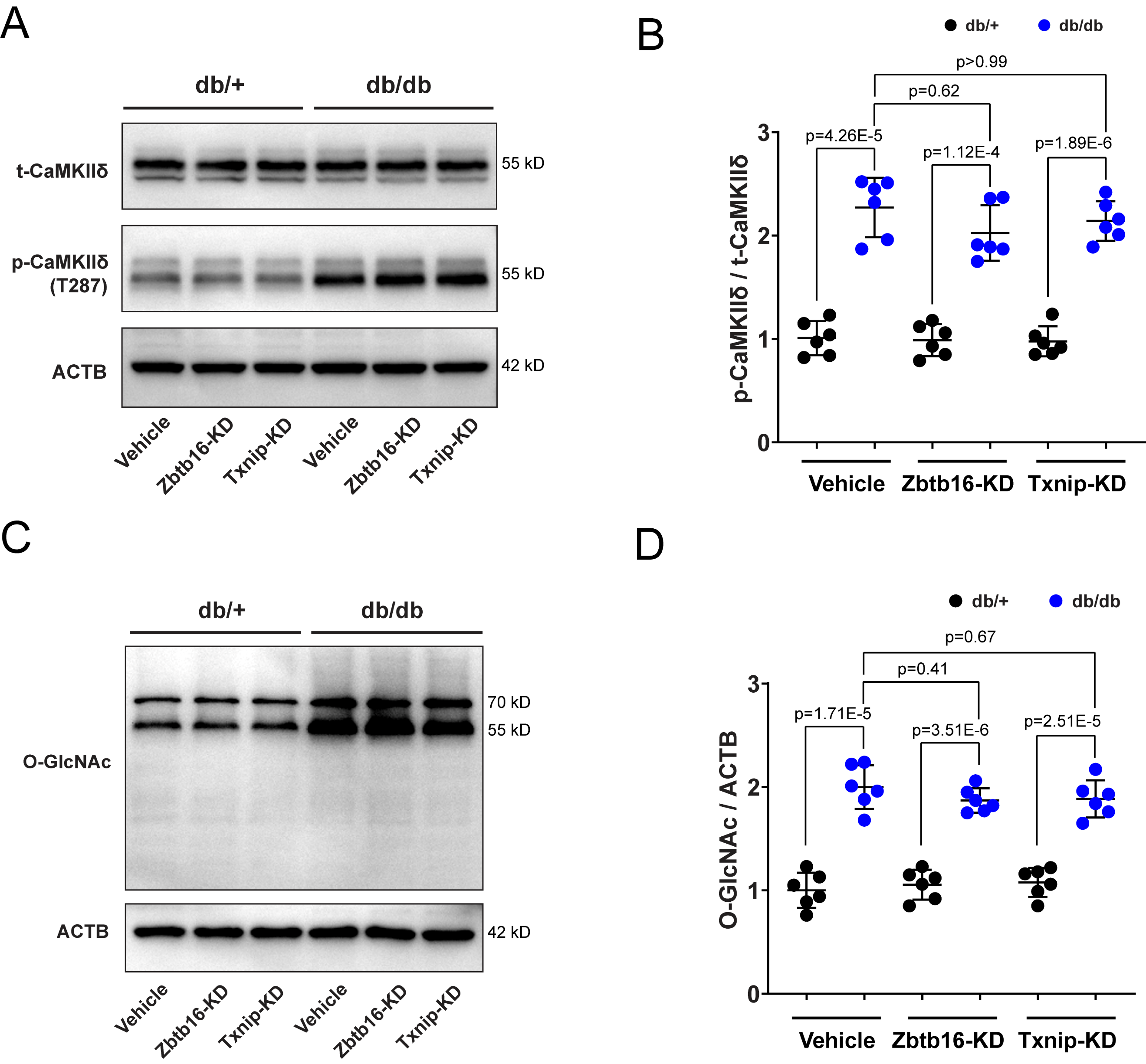


**Fig. S3** Expression of p-CaMKIIδ and O-GlcNAc in db/+ and db/db mouse atria. **A**, **B** Representative Western blot images (**A**) and quantitative analysis (**B**) of p-CaMKIIδ (n=6). **C**, **D** Representative Western blot images (**C**) and quantitative analysis (**D**) of O-GlcNAc (n=6). Data in (**B**, **D**) were analyzed by two-way ANOVA with Bonferroni’s multiple comparisons test. O-GlcNAc, O-GlcNAcylation


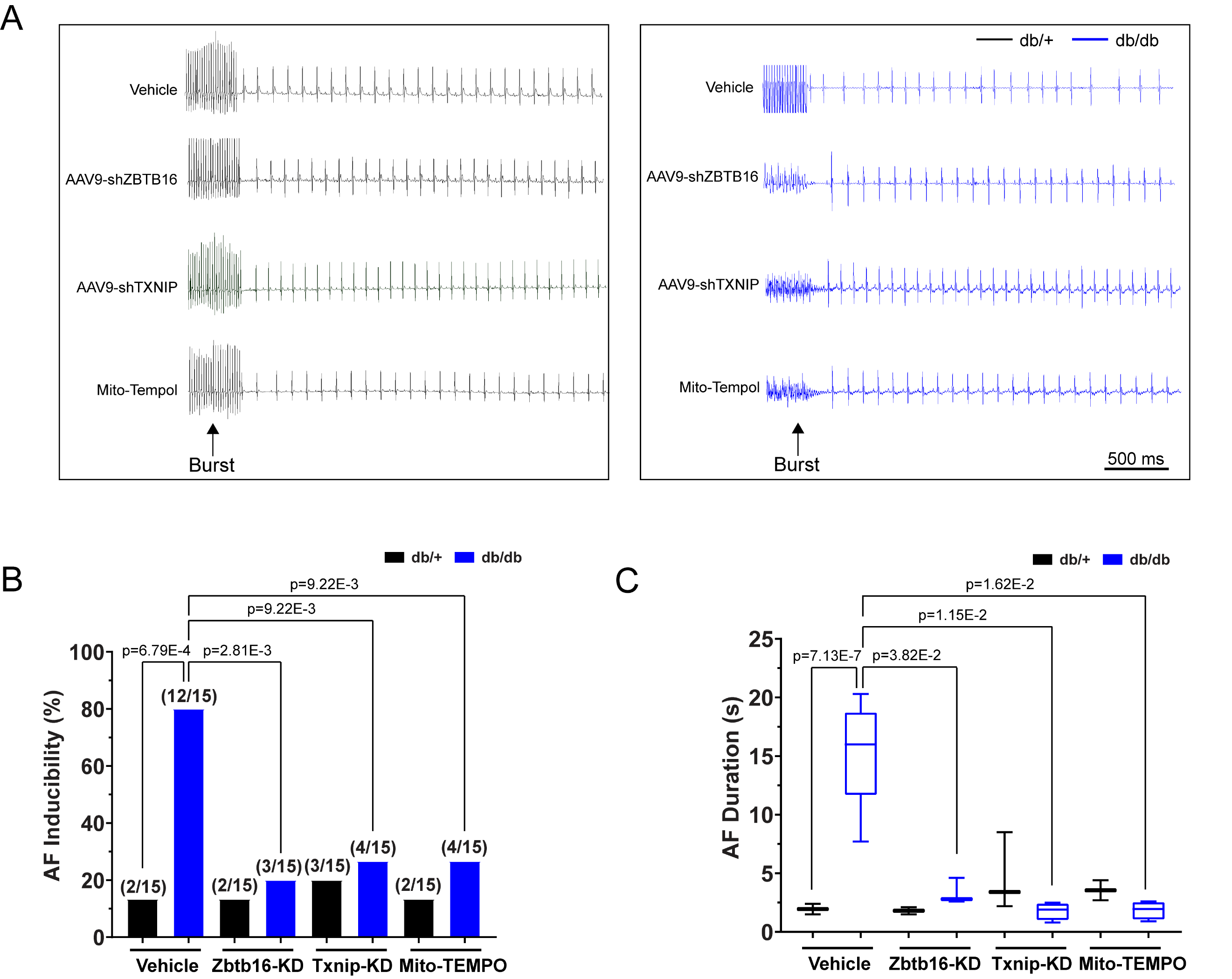


**Fig. S4** Intracardiac programmed electrical stimulation in db/+ and db/db mice. **A** Representative surface ECGs of db/+ and db/db mice induced by intracardiac electrical stimulation. Bar=500 ms. **B**, **C** AF inducibility (**B**, n = 15 for each group) and duration (**C**) analysis. Data in (**B**) were analyzed by Fisher’s exact test. Data in (**C**) were analyzed by two-way ANOVA with Bonferroni’s multiple comparisons test. AF, atrial fibrillation. KD, knockdown

**
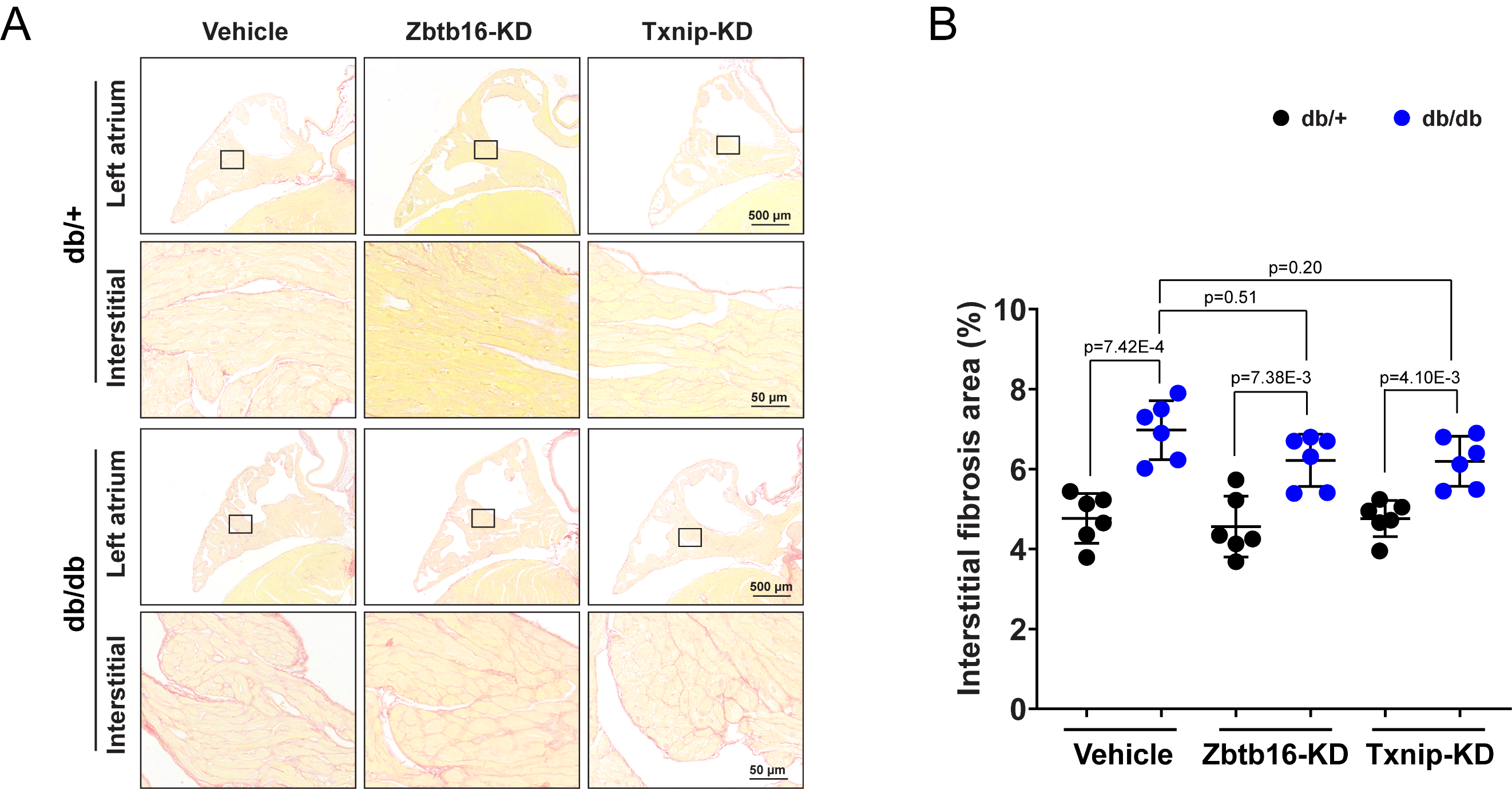
**

**Fig. S5** Area of atrial interstitial fibrosis in db/+ and db/db mice. **A**, **B** Representative picrosirius red staining of the left atrium sections (**A**) and quantitative analysis of interstitial fibrosis area (**B**) in db/+ and db/db mouse atria (n=6). Bar=500 μm in overall images and bar=50 μm in images amplified from the overall. Data in (**B**) were analyzed by two-way ANOVA with Bonferroni’s multiple comparisons test
